# Hyperactive and impulsive behaviors of LMTK1 knockout mice

**DOI:** 10.1038/s41598-020-72304-z

**Published:** 2020-09-22

**Authors:** Miyuki Takahashi, Arika Sugiyama, Ran Wei, Shizuka Kobayashi, Kimiko Fukuda, Hironori Nishino, Roka Takahashi, Koji Tsutsumi, Ichiro Kita, Kanae Ando, Toshiya Manabe, Hiroyuki Kamiguchi, Mineko Tomomura, Shin-ichi Hisanaga

**Affiliations:** 1 Laboratory of Molecular Neuroscience, Department of Biological Sciences, Graduate School of Science, Tokyo Metropolitan University, Minami-osawa, Hachioji, Tokyo 192-0397 Japan; 2 Division of Neuronal Network, Department of Basic Medical Sciences, Institute of Medical Science, The University of Tokyo, Tokyo, 108-8639 Japan; 3 Developmental Biology, Department of Biological Sciences, Tokyo Metropolitan University, Minami-osawa, Hachioji, Tokyo 192-0397 Japan; 4 Laboratory of Behavioral Neuroscience, Department of Human Health Sciences, Tokyo Metropolitan University, Minami-osawa, Hachioji, Tokyo 192-0397 Japan; 5 Laboratory for Neural Cell Dynamics, RIKEN Center for Brain Science, Wako, Saitama 351-0198 Japan; 6 Department of Oral Health Sciences, Meikai University School of Health Sciences, Urayasu, Chiba 279-9950 Japan; 7 Present Address: Department of Life Science and Medical Bio-Science, Waseda University, Shinjuku-ku, Tokyo, 162-8480 Japan; 8 Present Address: Division of Cell Biology, Department of Biosciences, School of Science, Kitasato University, Kanagawa, 252-0373 Japan

**Keywords:** Molecular neuroscience, Developmental disorders

## Abstract

Lemur tail kinase 1 (LMTK1), previously called Apoptosis-Associated Tyrosine Kinase (AATYK), remains an uncharacterized Ser/Thr protein kinase that is predominantly expressed in the brain. It is recently reported that LMTK1A, an isoform of LMTK1, binds to recycling endosomes through its palmitoylation and regulates endosomal trafficking by suppressing the activity of Rab11 small GTPase. In neurons, knockdown or knockout of LMTK1 results in longer axons, greater branching of dendrites and increased number of spines, suggesting that LMTK1 plays a role in neuronal circuit formation. However, its in vivo function remained to be investigated. Here, we examined the brain structures and behaviors of LMTK1 knockout (KO) mice. LMTK1 was expressed in most neurons throughout the brain. The overall brain structure appeared to be normal in LMTK1 KO mice, but the numbers of synapses were increased. LMTK1 KO mice had a slight impairment in memory formation and exhibited distinct psychiatric behaviors such as hyperactivity, impulsiveness and high motor coordination without social interaction deficits. Some of these abnormal behaviors represent core features of attention deficit hyperactive disorder (ADHD), suggesting the possible involvement of LMTK1 in the pathogenesis of ADHD.

## Introduction

Neuronal circuit wiring is the structural and functional basis of higher brain activities such as recognition and behaviors. To establish neuronal connections, neurons extend long processes of axons and dendrites, forming synapses between them, through which the neurons communicate with each other. For normal brain functions, it is important to accomplish the correct wiring between appropriate neurons. The formation and extension of axons, dendrites and spines (postsynaptic protrusions of excitatory synapses) accompany surface expansion, which requires a supply of membrane components, including both lipids and proteins. The supply of membrane components is mainly carried out by endosomal transport^1,2^. Particularly in highly polarized neurons, it is crucial to deliver membrane components to the appropriate places in the required amounts. The trafficking of endosomal vesicles is regulated by a family of Rab small GTPases^3–5^. There are more than 60 Rab family members, each of which has a distinct role in the respective membrane compartments. In comparison to the relatively well-described function of Rabs in nonneuronal cells, however, Rabs in neurons remain to be investigated. In particular, little is known about the regulatory mechanism of the respective Rabs in neurons.

Lemur tail (former tyrosine) kinase 1 (LMTK1) is, as yet, an uncharacterized protein kinase, which was previously called Apoptosis-Associated Tyrosine Kinase (AATYK) because it was isolated from apoptotic myeloid cells and showed amino acid sequence similarity in the kinase domain to receptor-type tyrosine kinase^6^. However, it was later shown to be abundantly expressed in neurons^7,8^ and thought to be a Ser/Thr kinase based on the Ser/Thr phosphorylation activity of its family kinase LMTK2^9,10^. All LMTK family members LMTK1 ~ 3 are large membrane-binding proteins composed of more than 1,300 amino acids, with a kinase domain in the N-terminal region and a long proline-rich C-terminal tail^11,12^. The C-terminal tail may serve as a scaffold for signaling proteins. For example, both LMTK1 and LMTK2 were isolated as a protein, which binds to the p35 Cdk5 activator^13,14^. LMTK2 was isolated as a myosin VI-binding protein^15,16^ and shown to bind to protein phosphatase-1 and Inhibitor-2^9,17^. These results support their role in the regulation of endosomal function, but their exact mechanism of action remains elusive, with only limited information available to date.

**Figure 1:**
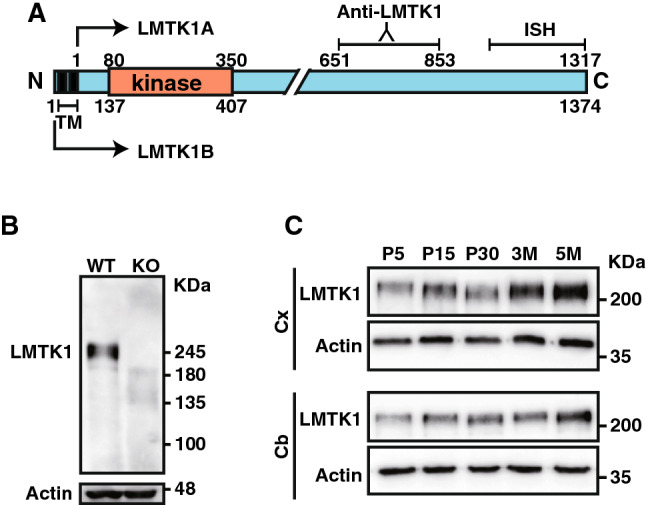
Expression of LMTK1 in mouse brain. (**A**) The molecular structure of LMTK1. LMTK1A and LMTK1B are alternative splicing variants of LMTK1 with the difference in the transmembrane sequence (TM) at the N-terminal end; LMTK1A does not, whereas LMTK1B does have the transmembrane sequences. LMTK1A and LMTK1B are composed of 1,317 and 1,374 amino acids, respectively, with a kinase domain (Kinase) in the N-terminal region. The probe used for in situ hybridization (ISH) was composed of 1,191 bases in the 3′-terminal coding region, and anti-LMTK1 was generated against the peptide of amino acids 651–853 of LMTK1A. (**B**) An immunoblot of WT and KO mouse brain with anti-LMTK1 antibody (upper). Actin served as the loading control (lower). (**C**) Expression of LMTK1 in cerebral cortex and cerebellum. Extracts of cerebral cortex (Cx) and cerebellum (Cb) of mouse brain from postnatal day 5 (P5) to 5 months (5M) were immunoblotted with anti-LMTK1. Actin served as the loading control. Uncropped immunoblots of LMTK1 and actin are provided in Supplementary Fig. 6.

LMTK1 consists of two isoforms of LMTK1A and LMTK1B, which are generated by alternative splicing. LMTK1A and LMTK1B are distinguished by the presence or absence of the N-terminal transmembrane sequences^12,18^ (see also Fig. 1A); LMTK1B contains the transmembrane sequences, whereas LMTK1A does not. Nevertheless, LMTK1A associates with endosomes through myristoylation at cysteine residues in the N-terminal region^19^. The trafficking of Rab11-positive recycling endosomes is negatively regulated by LMTK1A^20,21^. In neurons, LMTK1A negatively regulates axon elongation and dendrite arborization through activation of Rab11; knockdown or knockout of LMTK1 (*aatk*) results in increased neurite outgrowth^21,22^. However, its in vivo functions have not yet been analyzed.

While there is some, though limited, biochemical and cell biological data for LMTK1, almost nothing is known about its in vivo function. Only results obtained using mice lacking LMTK1 revealed higher dendritic arborization at early postnatal days of brain development^21^; however, behavioral analyses of knockout mice have not been conducted. In this study, we analyzed the expression of LMTK1 and the effect of its deficiency on brain structure and behaviors.

## Results

### LMTK1is ubiquitously expressed in mouse brain neurons

When brain lysates of wild type (WT) and LMTK1 knockout (KO) mice were blotted with anti-LMTK1, a single but slightly broad band was detected at a molecular mass of approximately 250 kDa in WT. While this size was much larger than that predicted from the amino acid sequences, 139.5 kDa and 145.5 kDa for the 1,317 and 1,374 amino acids of LMTK1A and LMTK1B (Fig. 1A), respectively, the band was shown to correspond to LMTK1 based on the absence in KO mouse brain (Fig. 1B). Immunoblots with anti-LMTK1 indicated its expression at postnatal day 5 (P5) and gradual increase with brain development in both the cerebral cortex (Cx in Fig. 1C) and cerebellum (Cb in Fig. 1C). We recently reported that the band is composed of two isoforms, which could not be distinguished by immunoblotting^18^.

LMTK1 was isolated as a protein bound to the p35 Cdk5 activator^13^, and was shown to modulate the trafficking of Rab11-positive endosomes^21,22^. Then, we examined expression levels of those LMTK1-related proteins in LMTK1 KO mouse brain by immunoblotting. However, there were no differences in the expression of these proteins between WT and LMTK1 KO mice (Supplementary Fig. 1).

**Figure 2:**
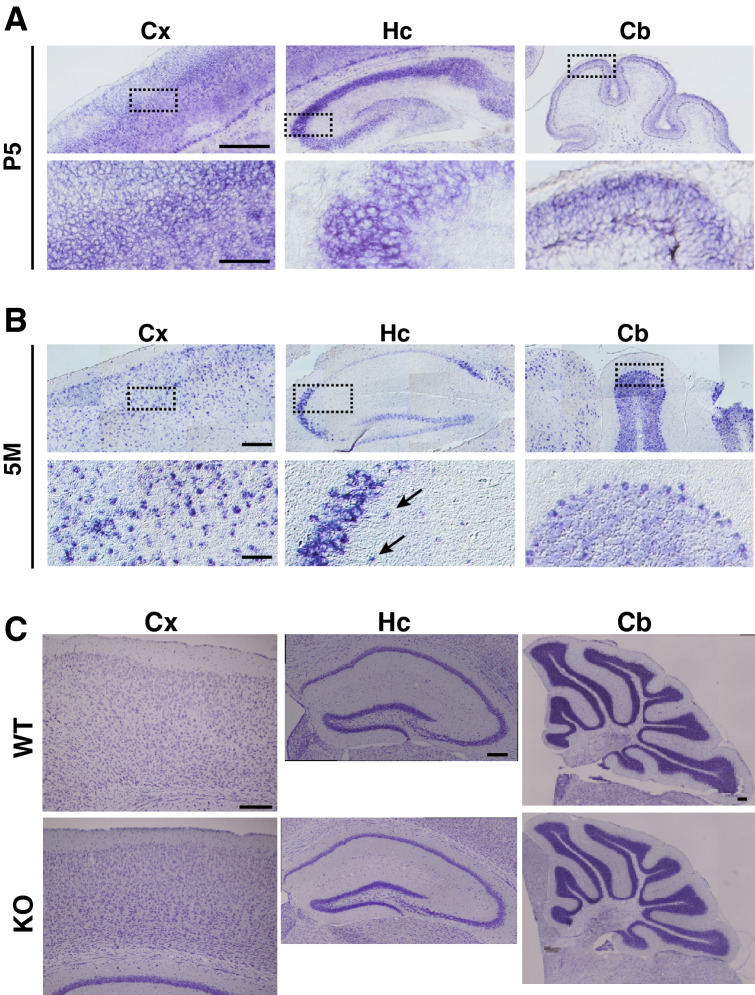
In situ hybridization of LMTK1 mRNA in mouse brain and structures of the LMTK1 KO mouse brain. (**A**) and (**B**) Expression of LMTK1 transcripts in cerebral cortex (Cx), hippocampus (Hc) and cerebellum (Cb) of mice at P5 (**A**) and 5M (**B**). Lower panels are higher magnification of the box shown in the upper panels. Arrows in the lower panel of Hc in (**B**) indicate nonneuronal cells labeled in white matter. Bar, 300 μm for the upper and 60 μm for the lower panels in both (**A**) and (**B**). (**C**) Structures of the LMTK1 KO mouse brain. Nissl staining of the cerebral cortex (Cx), hippocampus (Hc) and cerebellum (Cb) of the WT (upper) or KO (lower) mouse brain at 5M. Bars represent 200 µm.

The expression of LMTK1 was examined in brain regions. Because anti-LMTK1 could not be used for immunostaining of brains, we employed the in situ hybridization method to detect LMTK1 transcripts using 1,191 bases in the 3′-terminus-encoding region as a probe (Fig. 1A, ISH). The cerebral cortex, hippocampus and cerebellum at postnatal day 5 (P5) and at 5 months (5M) are shown in the panels of Fig. 2A,B, respectively. The region enclosed in the dotted box is magnified in the respective lower panel. LMTK1 signals were detected mainly in the cell body of most neurons in the cerebral cortex (Cx in Fig. 2A,B, left panels), hippocampus (Hc in middle panels) and cerebellum (Cb in right panels), indicating its prominent expression in neurons. However, signals were also found in cells scattered in the white matter (see, for example, the arrows in the lower middle panel of Fig. 2B), which might be oligodendrocytes as recently reported^23^. No signals were detected with a sense probe (Supplementary Fig. 2), demonstrating the specificity of the probe.

While LMTK1 was expressed in most neurons, there was no apparent anatomical abnormality in the KO mouse brain. All regions of the cerebral cortex (Cx), hippocampus (Hc) and cerebellum (Cb) appeared normal, similar to those of WT mouse, when they were observed by Nissl staining (Fig. 2C).

### Increased numbers of excitatory synapses in the brain of LMTK1 KO mouse

**Figure 3:**
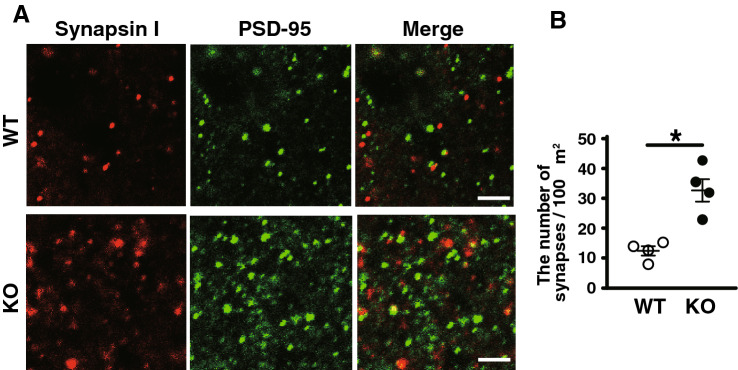
The number of synaptic staining in the cortex of LMTK1 KO mouse brain. (**A**) Immunostaining of cerebral cortex of WT and KO mice with anti-synapsin I (left) and anti-PSD-95 (middle). Merges are shown in the right. Bars represent 2 μm. (**B**) The number of synapses in KO mouse brain. Immunofluorescent puncta stained nearby or overlap with both synapsin I and PSD-95 were considered to be synapses and counted.

We recently report that the increase in the PSD-95-positive dots in the cerebral cortex of LMTK1 KO mouse brain^27^. Here, we confirmed it and examined whether the number of synapses is increased by coimmunostaining with antibodies to synapsin I presynaptic and PSD-95 postsynaptic proteins. Synapses were indeed increased in the cerebral cortex of LMTK1 KO mouse brain (Fig. 3, means ± SEM, n = 4 for both WT and KO mice, *P = 0.026, Mann–Whitney U-test). Because LMTK1 was expressed in most neurons throughout mouse brain (Fig. 2), we confirmed the effect of LMTK1 depletion on the number of synapses in cerebellum as another brain region. We observed the higher number of puncta stained with either VAMP-2 or synapsin I in the cerebellum of KO mice than in WT mice (Supplementary Fig. 3), suggesting that LMTK1 regulates the number of excitatory synapses in whole mouse brain.

We wondered if synaptic proteins are increased in the synaptic regions of LMTK1 KO mouse brain. To know it, we measured the amount of several synaptic proteins including NR2A and GluR2/3 glutamate receptors by immunoblotting using the crude synaptosomal fraction of LMTK1 KO mouse brain. However, the protein amounts of NR2A, GluR2/3, PSD-95 and synapsin I were not increased in LMTK1 KO mouse brain when normalized with actin (Supplementary Fig. 4), suggesting that the structure and function of synapses are not changed dramatically in LMTK1 KO mouse brain.

### Spatial and contextual fear conditioning is not markedly altered in LMTK1-KO mice

**Figure 4:**
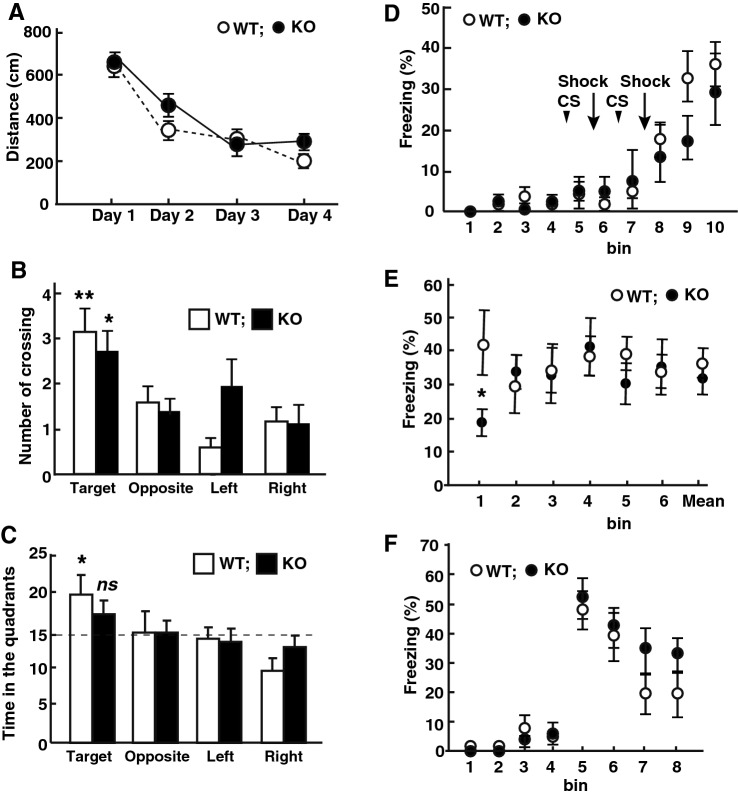
Effect of LMTK1 deficiency on memory formation. (**A**)–(**C**) Morris water maze test. (**A**) The total distance traveled by WT or KO mice to reach the platform during the training trials. (**B**) The probe test conducted 24 h after the final probe trial. The platform was removed, and the number of crossing the position of the target platform was counted. (**C**) The time mice spent in each quadrant was measured. The dashed line is the expected mean time. (**D**)–(**F**) Auditory and contextual fear conditioning test. (**D**) Freezing response during tone-shock condition trials. Arrows indicate the time point of foot shock stimuli after white noise (arrowheads, CS). (**E**) Freezing response during contextual fear conditioning performed in the same conditioning chamber. (**F**) Freezing response in the cued test before (trial number 1–4) and after (trial number 5–8) tone stimuli.

Because LMTK1 was expressed abundantly in the hippocampus (Fig. 2), we examined hippocampus-dependent spatial memory formation in LMTK1 KO mice using the Morris water maze. During training trials, KO mice swam the same distance as WT mice until reaching a hidden platform (Fig. 4A), indicating normal learning ability. In the probe trial, WT mice crossed more frequently the place where the platform was (Fig. 4B), and spent more time than the average in the quadrants where the platform was placed (Fig. 4C), as were expected. KO mice displayed the crossing number less than WT mice, but it was significantly different from the expected average (Fig. 4B, mean ± SEM, n = 10 for both WT and LMTK1 KO mice, **P < 0.001 and *P < 0.05 between the target and average, one sample *t* test). In contrast, while KO mice spent more time in the target quadrant than the expected average, it was not statistically significant (Fig. 4 C, mean ± SEM, n = 10 for both WT and LMTK1 KO mice, *P < 0.05 and ns, not significant between the target and average, one sample *t* test). These results appear to implicate a slight impairment of memory formation in LMTK1 KO mice.

We then examined associative learning by applying a contextual fear conditioning test using the electric foot shock and a tone as an unconditioned and conditioned stimulus, respectively. There was no significant difference in freezing time during conditioning between WT and KO mice (Fig. 4D). In the contextual fear conditioning, the freezing duration of KO mice was shorter than WT at the first bin but not at the following other bins (Fig. 4E, means ± SEM, n = 10 for both WT and LMTK1 KO mice, *P* = 0.0509, Mann–Whitney U-test). In the cued test, any difference between WT and KO mice was not detected (Fig. 4F, means ± SEM, n = 10 for both WT and LMTK1 KO mice, *p < 0.05, Mann–Whitney U-test).

### Basal electrophysiological synaptic activity is not changed in LMTK1 KO mice

**Figure 5:**
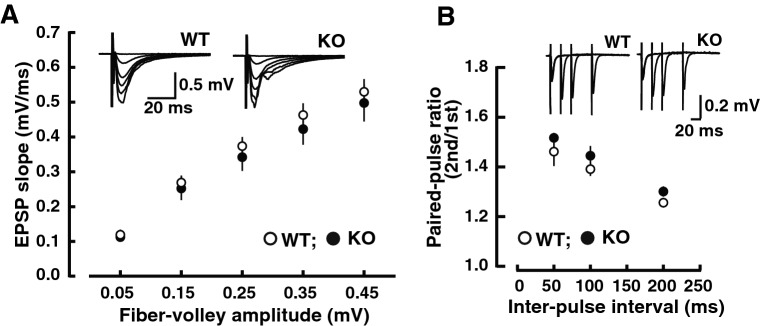
Basal synaptic transmission and paired-pulse facilitation in LMTK1 KO mice. (**A**) The input (fiber-volley amplitude)–output (EPSP slope) relationships of AMPA receptor-mediated EPSPs in WT (open circles, n = 8) and KO (filled circles, n = 9) mice. There was no significant difference between the two genotypes. Sample traces of EPSPs (average of 10 consecutive sweeps) evoked with various stimulus strengths are shown in the inset. (**B**) Paired-pulse facilitation (the ratio of slopes of the second EPSP to those of the first EPSP) shown as a function of interpulse intervals (IPIs) in the presence of 25 μM D-APV (WT, open circles, n = 9; KO, closed circles, n = 10). Right panel: sample traces of synaptic responses evoked by paired stimuli at intervals of 50, 100 and 200 ms are superimposed. All data are expressed as means ± SEM.

The increased number of synapses in LMTK1 KO mouse brains implicated higher synaptic activity in the KO mouse. Here, we evaluated the basal electrophysiological synaptic transmission in the CA1 region using hippocampal slices of mice at 10 to 16 weeks of age. However, there was no difference in the input (FV amplitude)-output (EPSP slope) relationship between WT and KO mice (Fig. 5A, representative traces are shown in the inset). We also examined paired-pulse facilitation (PPF), a presynaptic property demonstrating the increased synaptic response to the second pulse when two pulses are applied to afferent fibers at short intervals, such as tens to hundreds of milliseconds^24^. The PPF of KO mice was also identical to that of WT mice (Fig. 5B).

### LMTK1 KO mice show hyperactive behaviors and higher coordinated motor activity

**Figure 6:**
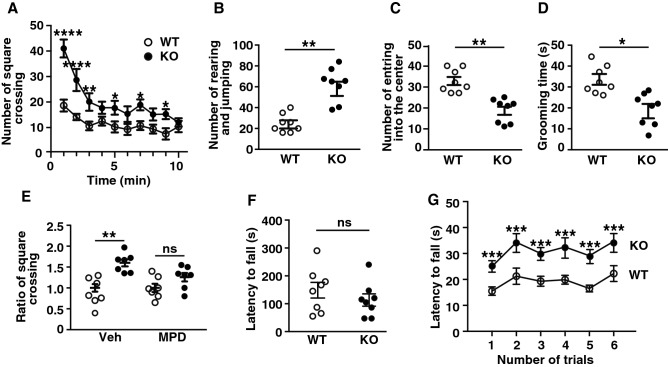
Hyperactivity of LMTK1 KO mice in an open field arena. (**A**) Horizontal activity. The frequency of square crossing in the open field arena separated into 9 squares was counted for 10 min of free movement. (**B**) Vertical activity. The frequency of rearing and jumping to the wall of an open field box. (**C**) The number of entry times into the center of the open field arena. (**D**) Grooming time. Time spent self-grooming was measured for 10 min. (**E**) Effect of methylphenidate (MPD) on horizontal activity in the open field arena. MPD was injected into mice at 0.05 mg/kg intraperitoneally, the movement of the mice was measured after 60 min by square crossing in the open field arena. PBS was injected as the control (Veh). (**F**) Wire hang test. Latency until falling was recorded manually, and the averages of two trials each per mouse are plotted. (**G**) Rotarod test. Mice were placed on a rod, which was rotated with a gradual increase in the rotation speed. The latency until falling off the rod was measured.

We noticed that LMTK1 KO mice struggle more than WT mice at the time of cage exchange. We speculated there might be some behavior abnormality in LMTK1 KO mice and subjected them to a battery of behavior tests. First, we observed the locomotive activity of LMTK1 KO mice using an open field test. When allowing them to move freely in the open field, KO mice exhibited a higher frequency of square crossing more than WT mice, not only immediately after they were placed but also after 5 min of acclimation to the environment (Fig. 6A, means ± SEM, n = 8 for both WT and LMTK1 KO mice, *p* = 0.0019 for genotypes, two-way repeated-measures ANOVA, *p < 0.05, **p = 0.0062, ****p < 0.0001, Sidak’s multiple comparison post hoc test), indicating that LMTK1 KO mice were more active. Interestingly, KO mice showed rearing or jumping to the wall two times more frequently than WT mice (Fig. 6B, means ± SEM, n = 8 for both WT and LMTK1 KO mice, ***p = 0.0009, Mann–Whitney U-test). In contrast, KO mice entered into the center of the open field arena less than WT mice (Fig. 6C, means ± SEM, n = 8 for both WT and LMTK1 KO mice, ***p = 0.0002, Mann–Whitney U-test) and engaged in a reduced duration of self-grooming than WT mice (Fig. 6D, means ± SEM, n = 8 for both WT and LMTK1 KO mice, **p = 0.0044, Mann–Whitney U-test). The hyperactivity of KO mice was suppressed by methylphenidate (MPD) (Fig. 6E, means ± SEM, n = 14 for both WT and LMTK1 KO mice, ****p<0.0001 for injection, two-way repeated-measures ANOVA, ns, not significant), a psychostimulant drug that is commonly used to treat hyperactivity and inattention in children diagnosed with attention deficit/hyperactivity disorder (ADHD)^25^. These results indicate that LMTK1 KO mice had a hyperactive and impulsive behavioral phenotype reminiscent of ADHD.

Next, we examined muscle strength by a wire hang test. However, no significant difference was detected in the latency until falling down between KO and WT mice (Fig. 6F, means ± SEM, n = 8, ns, not significant), indicating that hyperactivity was not simply due to physical strength. A rotarod performance test was employed to check motor coordination. LMTK1 KO mice showed higher performance at every session of 6 trials compared with WT mice (Fig. 6G, means ± SEM, n = 8 for both WT and LMTK1 KO mice, *p* < 0.0001 for genotypes, two-way repeated-measures ANOVA, ***p = 0.0001, Sidak’s multiple comparison post hoc test). Interestingly, the superior performance of KO mice was detected during the initial session of the trials, suggesting that the superior performance was not a result of training.

### No difference in social recognition ability between LMTK1 KO and WT mice

**Figure 7:**
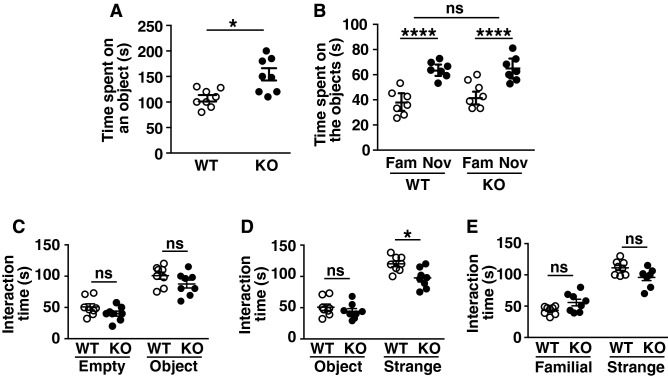
Novel object recognition and social interaction tests. (**A**) and (**B**) Novel object recognition test. A mouse was placed in the open field arena, where an object was placed. Time spent on the object was measured for 10 min (**A**). After acclimation with the object for 10 min, a novel object was placed, and the duration the mouse spent on the familiar object (Fam) and novel object (Nov) was measured for 10 min (**B**). (**C**)–(**E**) Social interaction test. After acclimation in the three-chamber box for 5 min, a test mouse was removed once from the box. An unfamiliar object (**C**), an object and strange mouse (**D**), or a familiar and strange mouse (**E**) were placed in one or both sections, and then the test mouse was returned to the arena. The interaction time was recorded for 10 min. The significant difference between WT and KO mice was obtained only with a strange mouse in (**D**). Others are not significant (ns).

When an object was placed in the center of the open field arena, KO mice spent more time on the object than WT mice (Fig. 7A, means ± SEM, n = 8 for both WT and LMTK1 KO mice, *p = 0.0351, Mann–Whitney U-test). We wondered if the LMTK1 deficiency might affect object recognition ability. To test this possibility, we placed another novel object and measured the time spent on each of them. While KO mice spent more time on the novel object than the old one, the duration was similar to that of WT mice (Fig. 7B, means ± SEM, n = 7 for both WT and LMTK1 KO mice, ****p<0.0001 for object two-way repeated-measures ANOVA, ns, not significant), suggesting that the LMTK1 deficiency did not affect object recognition ability.

We further carried out a social recognition test using a three-chamber box. When an object was placed in one chamber, both KO and WT mice spent more time in that chamber than the empty chamber (Fig. 7C, means ± SEM, n = 16 for both WT and LMTK1 KO mice, ns, not significant). When a strange mouse was placed in the empty chamber, both KO and WT mice spent a longer duration in the chamber of the strange mouse than the chamber of the object (Fig. 7D, means ± SEM, n = 16 for both WT and LMTK1 KO mice, *p = 0.0176, Sidak’s multiple comparison post hoc test).There was a significant difference between WT and KO mice in the chamber of strange mouse (*p* = 0.0176), but now we do not know whether this difference is derived from the recognition ability or simply due to the hyperactivity. Further, when both strange and familiar mice were placed in different chambers, KO mice spent a longer time in the chamber of the strange mouse than of the familiar mouse, but the durations were not different significantly from WT mice (Fig. 7E, means ± SEM, n = 16 for both WT and LMTK1 KO mice, ns, not significant). These results indicated that the lack of LMTK1 did not alter social recognition ability.

### Reduced anxiety and depression-like behavior in LMTK1-KO mice

**Figure 8:**
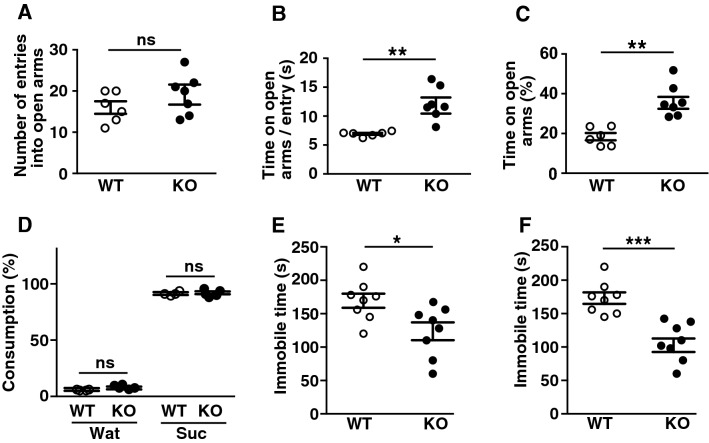
Anxiety and depression tests. (**A**)–(**C**) Elevated plus maze test. Mice were placed on the center of the elevated platforms with two open and two closed arms. Entry into the open arms (**A**), time spent on the open arm per entry (**B**) and total time on the open arms (**C**) were measured. (**D**) Sucrose preference test. Mice were acclimated with 2 bottles for 4 days. On days 5–8, the mice were presented with 2 bottles, one containing water (Wat) and one sucrose (Suc). Consumption was measured daily. (**E**) Tail suspension test. The immobility time for the last 5 min of the 6-min test was measured. (**F**) Forced swimming test. The immobility time of mice placed in a swimming pool for the last 5 min of the 6-min test duration.

LMTK1 KO mice entered the center of the open field arena less frequently than WT mice (Fig. 6C). We speculated that this result was a consequence of the increased time spent around the wall of the open field arena, but it was also possible that KO mice experienced less anxiety than WT mice. To test the anxiety-like behavior of LMTK1 KO mice, we employed an elevated plus maze test (Fig. 8A–C). When the mice were placed on the center of the elevated plus maze, the KO mice entered the open arms at a similar frequency to WT mice (Fig. 8A). However, because they spent significantly more time on the open arms than WT mice (Fig. 8B), the total time on the open arm was longer for KO than WT mice (Fig. 8C), suggesting less anxiety of LMTK1 KO mice (Fig. 8A–C, means ± SEM, n = 6 for WT and n = 7 for LMTK1 KO mice, *p* = 0.4610 for A, **p = 0.0017 for B, **p = 0.0012 for C, Mann–Whitney U-test, ns, not significant).

Subsequently, we examined the anti-depressant-like behavior of LMTK1 KO mice. First, we tested the basal depressant condition by a sucrose preference test and found no difference in intake preference between WT and KO mice (Fig. 8D, means ± SEM, n = 8 for WT and n = 10 for LMTK1 KO mic, ns, not significant). Next, we subjected KO mice to the tail suspension (Fig. 8E) and forced swimming tests (Fig. 8F). Under inescapable stress conditions in these tests, the immobility time is considered indicative of depression behavior, which was measured during stress loading. In the tail suspension test, the immobility time was significantly shorter in KO compared with WT mice, with an immobility time of approximately 150 s (Fig. 8E, means ± SEM, n = 8 for both WT and LMTK1 KO mice, *p = 0.0402, Mann–Whitney U-test)^26^. In the forced swimming test, the immobility time of KO mice was also less than WT mice (Fig. 8F, means ± SEM, n = 8 for both WT and LMTK1 KO mice, ***p = 0.0008, Mann–Whitney U-test). These results suggest that LMTK1 KO mice feel depressant stress less than WT mice.

## Discussion

LMTK1 is a poorly characterized Ser/Thr protein kinase that is expressed highly in the brain. It is previously reported using cultured primary neurons that LMTK1 regulates the outgrowth of axons, arborization of dendrites and spine formation via membrane trafficking^21,22,27^. However, the in vivo function of LMTK1 remained to be investigated. Here we examined the brain structures and behaviors of LMTK1 KO mice, and we observed an increase in synapses in the cerebral cortex, while the overall brain structures appeared normal. LMTK1 KO mice exhibited abnormal behaviors such as hyperactivity, impulsive jumping, high motor coordination, less anxiety and anti-depressant behaviors, some of which are reminiscent of ADHD, implicating the involvement of LMTK1 in ADHD-related hyperactive behaviors.

LMTK1 is a member of the LMTK Ser/Thr kinase family. There are three LMTKs, LMTK1 ~ 3 with two splicing variants in LMTK1, LMTK1A and LMTK1B^12,18^. All of them are highly expressed in the brain^10,12,14,28^ and associate with endosomes through the two transmembrane sequences at the N-terminus of LMTK1B, LMTK2 and LMTK3^12,29^, or through palmitoylation of cysteine residues at the N-terminus of LMTK1A^19,20^. The association with endosomes suggests their roles in endosomal functions^15,16^. In fact, LMTK1A binds to Rab11-positive recycling endosomes and regulates endosome trafficking in axons, dendrites and spines^19–21,27^. However, the molecular mechanism of its action is not known. Furthermore, physiological substrates are also unknown not only for LMTK1 but also other LMTKs, which is, at least partly, due to their marginal kinase activity. Although LMTK2 has been reported to have kinase activity in vitro by, for example, phosphorylation of cystic fibrosis transmembrane conductance regulator (CFTR) and phosphorylase b^30^, the kinase activity of LMTK1 and LMTK3 is suggested only by their autophosphorylation^8,10^. Gene knockout is a promising strategy to approach the in vivo function of LMTK kinases. Indeed, KO mice of LMTK2 and LMTK3 have already been reported. While LMTK2 KO mice show no morphological brain abnormalities, they exhibit impaired spermatogenesis with defects in morphological differentiation to elongated spermatids^31^. It is known that the testis and brain frequently express similar types of genes and that sperm tail elongation requires a membrane supply^32–35^. LMTK3 KO mice do not show histological abnormalities in the brain but exhibit behavioral abnormalities such as locomotor hyperactivity and reduced anxiety^28^. The intracellular levels of GluN1 and GluN2 glutamate receptors are biochemically shown to increase in LMTK3 KO mouse brains. During the preparation of the manuscript, further analysis of the LMTK3 KO mouse was reported^36^. According to that report, LMTK3 KO mice exhibit memory and cognitive dysfunction and are proposed as a mouse model of schizophrenia and bipolar disorders. The present study is the first characterization of LMTK1 KO mice. Gene depletion of LMTK1 (*aatk*) did not affect the overall structure of the brain as reported for KO mice of LMTK2 or LMTK3, but we found the increase in the number of synapses in the cortex and cerebellum of adult KO mice. This phenotype can be explained by the increased supply of membrane components to the pre- and post-synaptic regions. Further, LMTK1 KO mice exhibited hyperactivity like LMTK3 KO mice. While information is still fragmented, these results altogether suggest that LMTK family kinases contribute to proper psychiatric activities by regulating the delivery of synaptic membrane proteins.

Although LMTK kinases appear to share similar functions in endosomal trafficking, the mechanisms are largely unknown. Here, it is worthy to discuss the factors upstream and downstream of LMTK1. LMTK1 is isolated as a protein bound to p35 Cdk5 activator by yeast two hybrid screening^13^, is phosphorylated at Ser34 by Cdk5-p35^37^, and is found that the phosphorylation regulates LMTK1 activity^21^. LMTK2 is also isolated as a p35 binding protein^14^ and is phosphorylated at Ser1418 by Cdk5-p35^17^. We also observed phosphorylation of LMTK3 by Cdk5-p35 (Supplementary Fig. 5), indicating the close association of LMTK family members with Cdk5-p35. Interestingly, the kinase active Cdk5 binds to endosomal vesicles through myristoylation of p35^38,39^, indicating their close proximity on the cytoplasmic surface of endosomes. Mice lacking the p35 gene (*cdkr1*) have been reported to display a hyperactive phenotype and less anxiety^40,41^. The hyperactivity was further reversed to normal level by treatment with psychostimulants, as observed for LMTK1 KO mice treated with MPD. These reports suggest that the absence of p35 results in reduced Cdk5 activity, leading to the dysregulation of dopaminergic transmission^40,41^. Cdk5-p35 is a multifunctional protein kinase that phosphorylates cytoskeletal and signaling proteins as well as proteins involved in membrane trafficking^38,42–44^. p35 KO induces the structural abnormality of brains such as the inverted layers of cortical neurons^45^, which differs from LMTK1 KO mice. The present results suggest that the hyperactive behavior can be induced without the structural abnormality of the cortical layers. Among multiple Cdk5 functions, LMTK1 may play a role in a part of the membrane transport, in which case, it would be interesting to explore the Cdk5-LMTK1 axis as a possible signaling pathway involved in hyperactive behaviors.

Regarding the events downstream of LMTK1, it is previously reported that LMTK1 regulates endosomal transport in axons and dendrites of primary neurons by suppressing Rab11 activity^21,22^. Further, it is recently shown that TBC1D9B, a protein with Rab11 GAP activity, mediates the LMTK1 signal to Rab11^27^. Therefore, we hypothesize that the TBC1D9B-Rab11-recycling endosome is a downstream cascade of LMTK1, which controls axon and dendrite outgrowth and spine density through the supply of membrane components. Based on the previous findings that the knockout or knockdown effects of LMTK1 on axon outgrowth and dendrite branching were so remarkable in cultured neurons, we anticipated that there would be brain structural abnormalities in LMTK1 KO mice, but this was not the case. Overall structural abnormalities were not observed, but instead we detected microscopic changes in the number of synapses of the LMTK1 KO mouse brain. Even though these changes were induced, the basal electrophysiological activity was not affected. Similar results have been obtained with LMTK3 KO mice^28^. At present, we do not know the cause, but the effect of LMTK1 KO on behavior could be derived from events that occur during the developmental stages of the brain. In fact, increased dendrites of hippocampal neurons at P7, but not in the adult, were observed by silver impregnation^21^. If the speed of axon outgrowth is increased, the axon would mistarget to different neurons from those genetically programmed. If dendritic arborization is enhanced, neurons would receive more synaptic input from other neurons than normal neurons. The improper establishment of neuronal connections would perturb psychiatric behavior in adulthood, even though most of the improper connections would be gradually corrected in an activity-dependent manner during brain maturation.

LMTK1 KO mice exhibited a number of behavioral abnormalities. In particular, remarkable features of LMTK1 KO mice were locomotive hyperactivity, impulsive jumping against the wall of the open field arena and climbing the object in the open field, which are known as core phenotypes of ADHD^46^. By contrast, LMTK1 KO mice also exhibited higher motor coordination performance in the rotor rod test, which are not categorized as symptoms of ADHD. Further, LMTK1 KO mice displayed reduced anxiety and anti-depressant behaviors, which are also not representative of ADHD. However, some behavioral abnormalities, including reduced anxiety and anti-depressant behavior, might be secondary results caused by hyperactivity. ADHD is a composite disorder caused by a number of genetic, epigenetic and environmental factors. Like many mutant mice that exhibit hyperactivity, a portion of the ADHD-related behaviors has been proposed as a model of ADHD. LMTK1 KO mice would also provide a model for investigating hyperactivity and impulsiveness among ADHD-related behaviors.

## Materials and methods

### Antibodies and chemicals

Anti-actin (A2066) was purchased from Sigma-Aldrich (St. Louis, MS). Anti-synapsin I (Ab64581) was obtained from abcam (Tokyo, Japan). Anti-PSD-95 (MA1-045) and anti-Rab11 were from Thermo Fisher Scientific (Grand Island, NY). Anti-p35 (C-19) and anti-Cdk5 (DC-17) were purchased from Santa Cruz Biotechnology (Santa Cruz, CA). Anti-GluR2/3 was from Millipore (Burlington, MA). Anti-NR2A antibody was purchased from Chemicon (Temecula, CA). Anti-VAMP-2 was provided by Dr. M. Itakura at Kitasato University. Anti-LMTK1 was generated by immunizing rabbits with the LMTK1 peptide of amino acids 651–853 (Fig. 1B). AlexaFluor 488-conjugated anti-mouse (AB_2556548) and AlexaFluor 568-conjugated anti-rabbit IgG (AB_143157) were obtained from Thermo Fisher Scientific. HRP-conjugated anti-mouse (AB_2617137) and anti-rabbit antibodies (AB_2617138) were from Agilent Dako (Santa Clara, CA). Picrotoxin and methylphenidate were purchased from Sigma-Aldrich. d-(-)-2-amino-5-phosphonovaleric acid (D-APV) and 6-cyano-7-nitroquinoxaline-2,3-dione (CNQX) were purchased from Tocris Bioscience (Avonmouth, UK).

### Animal experiments

Pregnant female mice of C57BL/6J were obtained from Crea Japan (Tokyo, Japan). ICR mice were obtained from Sankyo Laboratory Service (Tokyo, Japan). LMTK1 knockout (KO) mouse was described previously^21^. The mice were housed in a controlled environment at 22–25 °C and 50% humidity under a 12-h light/12-h dark cycle with free access to food and distilled water available ad libitum.

### In situ hybridization and immunocytochemistry

In situ hybridization was performed on 10-µm cryosections with a digoxigenin-labeled probe. Materials and reagents were all RNase free. An 1,191-bp 3′-terminal fragment of the LMTK1A cDNA coding sequence was used as a probe (Fig. 1A). The amplified PCR product was purified with the FavorPrep GEL/PCR Purification Kit (Favorgen Biotech Corp., Ping-Tung, Taiwan)**.**

The brains of mice at postnatal day 5 (P5) and 5 months (5M) after birth were fixed with 4% paraformaldehyde in phosphate-buffered saline (PBS) by perfusion. Frozen sections were generated with a Cryostar NX70 (Thermo Fisher Scientific). Specimens were stained with 0.1% cresyl violet in 10% acetic acid or fluorescently with anti-PSD-95, anti-VAMP-2 or anti-synapsin I antibodies. Images were acquired with a BZ-9000 (Keyence, Osaka, Japan) or an EXCITER or LSM 710 confocal microscope (Carl Zeiss, Jena, Germany).

### Preparation of brain extracts, SDS-PAGE and immunoblotting

The brains of mice at P5, P7, P30, 3M and 5M were homogenized in 20 mM HEPES (pH 7.5), 0.1 mM EDTA, 2 mM MgCl_2_, 5 mM KCl, 0.1 mM EGTA, 1 mM dithiothreitol, 0.2 mM Pefablock, and 1 μg/ml leupeptin, using a Teflon-pestle motor-driven homogenizer, and the extracts were obtained by centrifugation at 24,000×*g* for 20 min. The cerebral cortex or hippocampus of mouse brains at 5M was homogenized in 10 volumes of HEPES buffer (5 mM HEPES, pH 7.5, 1 mM EGTA, 1 mM dithiothreitol, 0.2 mM Pefablock, and 1 μg/ml leupeptin) as described above. The homogenates were centrifuged at 800×*g* for 10 min. The supernatant (S1) was centrifuged again at 10,000×*g* for 15 min. The pellet (P2) was suspended in HEPES buffer containing 0.32 M sucrose and centrifuged at 10,000×*g* for 15 min to yield the supernatant (S2′) and pellet (P2′). S2′ was used as the brain extracts and P2′ was used as the crude synaptosomal fraction. SDS-PAGE and immunoblotting were performed using 6% or 7.5% polyacrylamide gel^47^. All biochemical and cell biological experiments were performed at least three times, and representative results are shown.

### Electrophysiological analyses

Hippocampal slices were prepared from adult male mice aged 10 to 16 weeks. Synaptic responses were recorded in the stratum radiatum of the CA1 region. To evoke synaptic responses, Schaffer collateral/commissural fibers were stimulated at 0.1 Hz. The input–output relationship was examined in the presence of a low concentration of the non-NMDA receptor antagonist CNQX for accurate measurements. EPSPs were evoked with various strengths of stimulation, and the data were first sorted by binning the fiber volley amplitudes, followed by averaging of the EPSP slopes within each bin.

### Mouse behavior experiments

Behavior tests were conducted with adult male mice at 7–13 weeks of age, using either LMTK1 KO mice or WT littermates according to previously described methods^48–51^ Behavior experiments were carried out during the light-phase on the same day after mice were acclimated to a test room for 1 h prior to testing. Mouse behaviors were recorded using a video camera (JVC, Kenwood Corporation, Yokohama, Japan) and analyzed manually.

*Morris water maze test* Mice were trained to find the hidden platform in a circular pool for four consecutive days. After the last training, a probe test was performed using the pool from which the platform had been removed. The swim distance, time spent in the target quadrant zone and other quadrants, and number of times the position of the target platform was crossed were measured.

*Fear conditioning test* The mouse was placed in a chamber equipped with a stainless floor connected to an electric shock generator. The conditioning trial consisted of an exploration period followed by two conditioned stimulus (CS, auditory cue)—unconditioned stimulus (US, foot-shock) pairings. A context test as performed in the same conditioning chamber in the absence of CS. The cued test was performed in a different chamber with an exploration period followed by the CS period. The freezing response of the mice was measured.

*Open field test* The open field arena consisted of a 50 × 50-cm-wide and 50-cm-high white box with the floor divided into 9 equal squares. The mouse was placed in the center of the arena at the start of each trial and allowed to explore freely. The numbers of crossing lines between squares, entry into the center, rearing (standing on the hind legs) and jumping to the wall were counted during a 10-min session.

*Wire hang test* The mouse was placed on a wire mesh, set up 30 cm above the floor, and the wire mesh was then turned upside down. Time was recorded until the mouse fell off or the tests were ended at 5 min. Tests were repeated three times for each mouse.

*Rotarod performance test* Mice were placed on a rotarod apparatus (4 cm in diameter) for a 5-min training session at 6-rpm rotation. After the training session, the rotating speed was steadily increased to 60 rpm, and the latency to falling off was recorded for each mouse for two sessions with a 30-min interval.

*Novel object recognition test* The mouse was allowed to explore two identical objects freely in a test box for 10 min. Then, the mouse was removed from the test box and placed back in the home cage for 1 h. The mouse was carefully placed again in the test box, in which the familiar and novel objects were placed, and allowed to freely investigate the objects. Time interacting with the familiar or novel object was determined.

*Social interaction test* A test mouse was acclimated in the center of three-chamber box separated by two clear plexiglasses for 5 min. The mouse was removed from the box, and a novel object or a strange mouse and object were placed in one or both chambers. The test mouse was returned to the chamber, and the interaction time was recorded for 10 min. After the interaction test with the object and mouse, the object was replaced with another novel mouse, and the interaction time was again measured for 10 min.

*Elevated plus maze test* The mouse was placed on the center of an elevated platform apparatus consisting of two open and two closed arms. The number of entries and total time spent in the open arms, respectively, were quantified during a 10-min period.

*Sucrose preference test* The test mice were singly housed and provided with 2 identical bottles for drinking. The mice were acclimated to the 2 bottles for 4 days, both of which contained water on days 1 and 3, and 4% (w/v) sucrose solution on days 2 and 4. On days 5–8, the mice were presented with 2 bottles, one with water and one with sucrose. The consumption of water and sucrose was measured daily, and the bottle position was altered to avoid potential side bias as previously described^48^.

*Tail suspension test* The mice were suspended 50 cm above the floor by the tail via an adhesive tape at approximately 1 cm from the tip of the tail. The immobile periods were quantified during the last 5 min of the 6-min test.

*Forced swimming test* The forced swimming test was carried out in a 2-L beaker (15 cm in diameter, 20 cm in height) containing water at 25 ± 1 °C to a height of 17 cm. The duration of immobility was counted during the last 5 min of the 6-min test. Immobility was judged when mice ceased struggling and remained floating motionless in the water only with necessary movements to keep their heads above water.

### Data analysis

All data are presented as the means ± SEM. All comparisons of behavior experiments were between littermates. Statistical analyses of the open field, rotarod performance, novel object and social interaction tests were performed using two-way repeated-measures ANOVA followed by Sidak’s multiple comparison post hoc test using GraphPad Prism 6 (GraphPad Software, CA, USA). Electrophysiological data were analyzed on-line and off-line using pClamp software (Molecular Devices). Data for other behavior tests were analyzed using the Mann–Whitney U-test.

### Ethical statement

The study was approved by the Research Ethics Committee of Tokyo Metropolitan University (approval numbers; A30-1 and A31-24). All animal experiments were performed according to the guidelines for animal experimentation of Tokyo Metropolitan University (in accordance with the Society for Neuroscience Guidelines). All efforts were made to reduce the suffering of the animals.

## Supplementary information

Supplementary Information.

## Abbreviations

AATYK1: Apoptosis-Associated Tyrosine Kinase
ADHD: Attention deficit hyperactive disorder
Cx: Cerebral cortex
Cb: Cerebellum
CS: Conditioned stimulus
ISH: In situ hybridization
KO: Knockout
LMTK: Lemur tail kinase
PPF: Paired-pulse facilitation
US: Unconditioned stimulus
WT: Wildtype
