# Supplementary material for: Hyperactive and impulsive behaviors of LMTK1 knockout mice
Source: Sci Rep. 2020 Sep 22;10:15461. doi: 10.1038/s41598-020-72304-z (PMC7508861; doi:10.1038/s41598-020-72304-z)
Supplement: Supplementary file 1 — Supplementary Information. [file 41598_2020_72304_MOESM1_ESM.pdf]

Supplementary figures

Hyperactive and impulsive behaviors of LMTK1 knockout mice

Miyuki Takahashi, Arika Sugiyama, Ran Wei, Shizuka Kobayashi, Kimiko Fukuda, Hironori Nishino, Roka Takahashi, Koji Tsutsumi, Ichiro Kita, Kanae Ando, Toshiya Manabe, Hiroyuki Kamiguchi, Mineko Tomomura, and Shin-ichi Hisanaga

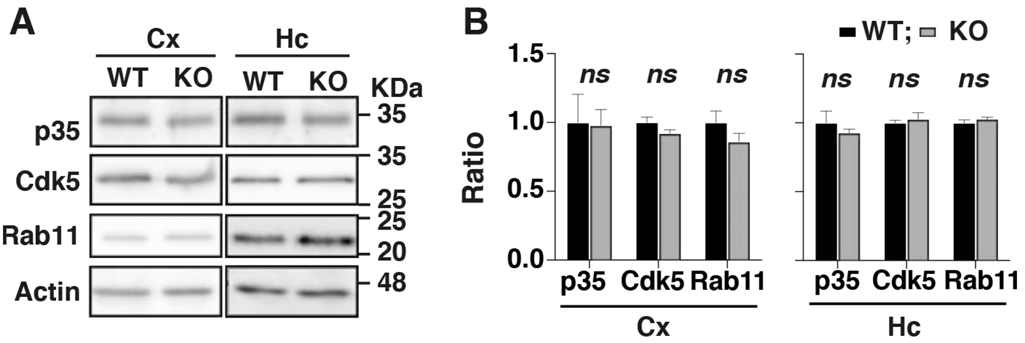

Supplementary Fig. 1. Expression levels of p35, Cdk5 and Rab11 in LMTK1 KO mouse brain. (A) Immunoblots showing the expression of p35, Cdk5 and Rab11 in LMTK1 KO mouse brain. The extracts of the cerebral cortex (Cx) and hippocampus (Hc) of WT and LMTK1 KO mouse brain at 5 months (5M) were immunoreacted with the antibody against p35, Cdk5 and Rab11. Actin served as the loading control. (B) Quantification of the expression levels (means ± SEM, n = 3, *ns*, not significant). Original uncropped immunoblots are provided in Supplementary Fig. 7, a ~ d.

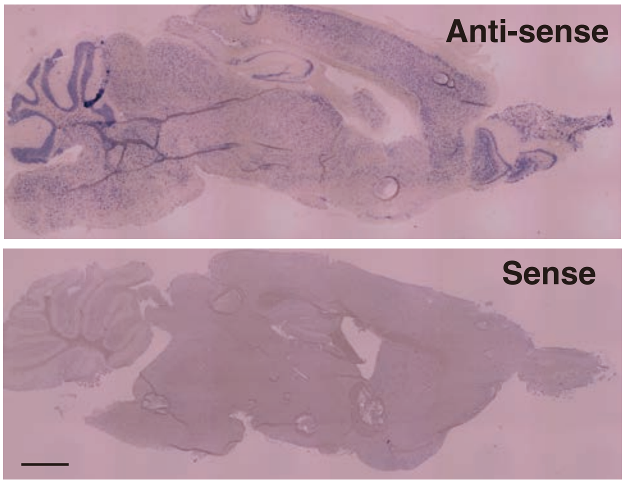

Supplementary Fig. 2. *In situ* hybridization showing LMTK1 mRNA expression in mouse brain. A sagittal cryosection of the whole brain of an adult mouse was hybridized with an antisense probe (upper) or a sense probe (lower) specific for LMTK1. The probe is 1000 ntd (Fig. 1A). Bar, 1000 μm.

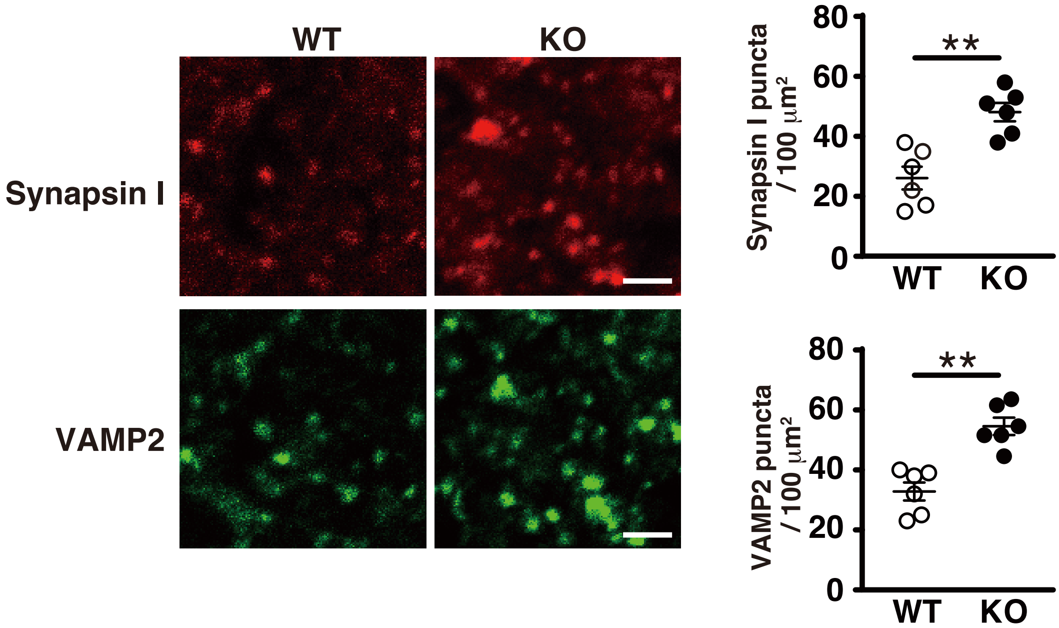

Supplementary Fig. 3. Presynaptic puncta in brains of WT and LMTK1 KO mouse. Cerebral cortex of mouse brain, either WT (left) or LMTK1 KO (right), was immunostained with anti-synapsin I (upper) and anti-VAMP-2 (lower). Bar, 2 μm. The number of presynaptic puncta was counted and expressed for anti-synapsin I (means ± SEM, n = 6 for both WT and LMTK1 KO mouse, ***p* = 0.0043, Mann-Whitney U-test), and for anti-VAMP-2 (means ± SEM, n = 6 for KO and n = 6 for LMTK1 KO mouse, ***p* = 0.0022, Mann-Whitney U-test).

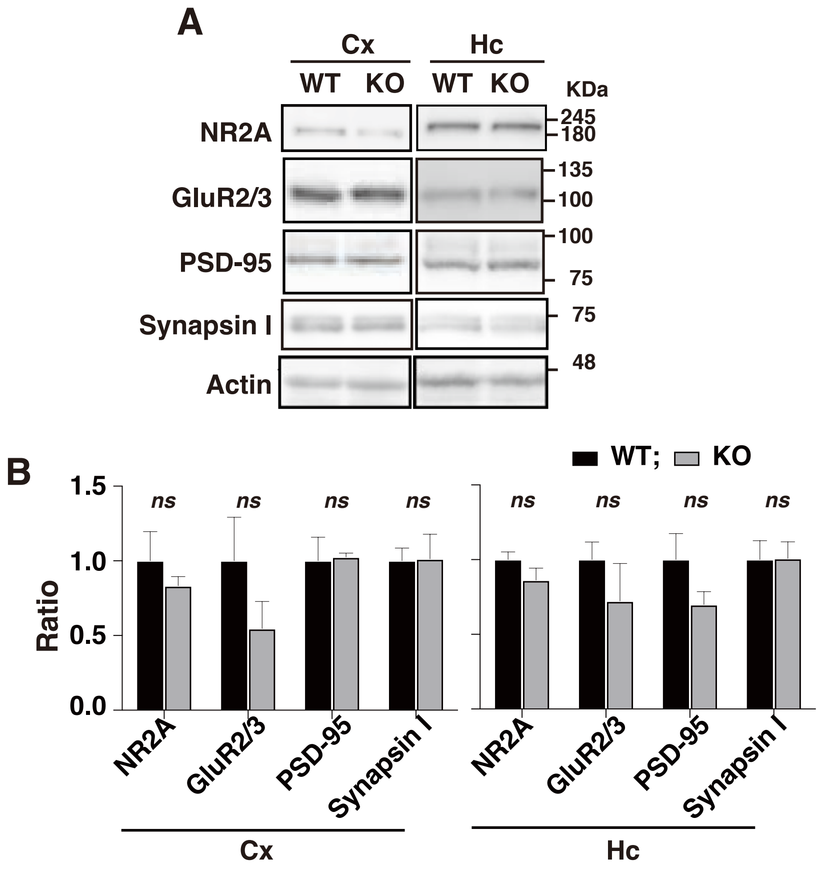

Supplementary Fig. 4. Expression levels of synaptic proteins in LMTK1 KO mouse brain. (A) Immunoblots of the crude synaptosomal fraction prepared from cerebral cortex (Cx) or hippocampus (Hc) of WT and LMTK1 KO mouse brains with the antibody against NR2A, GluR2/3, PSD-95 and synapsin I. Actin served as the loading control. (B) Quantification of the expression levels (means ± SEM, n = 3, *ns*, not significant). Original uncropped immunoblots of NR2A, GluR2/3, PSD-95, synapsin I and actin are provided in Supplementary Fig. 7, e ~ i.

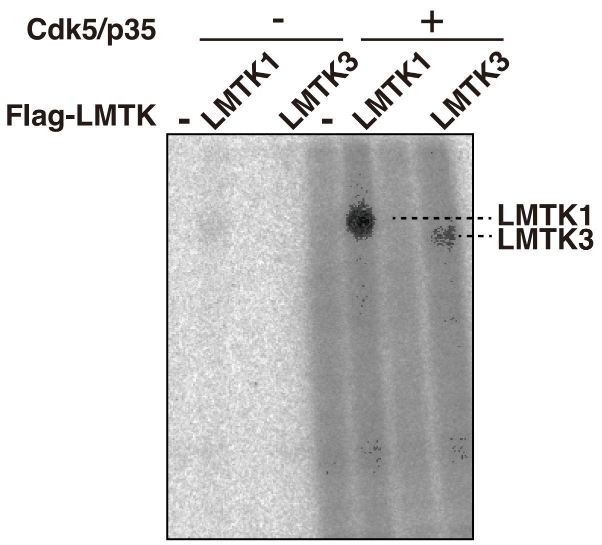

Supplementary Fig. 5. Phosphorylation of LMTK3 by Cdk5-p35. LMTK1A-Flag and LMTK3-Flag were expressed in HEK293 cells. After immunoprecipitation with anti-Flag antibody, they were phosphorylated by Cdk5-p35 in the presence of [γ–^{32}P]ATP. Phosphorylation was detected by autoradiograph using a BAS2000 Bioimage analyzer (GE Healthcare). Positions of LMTK1 and 3 are indicated by dotted lines. Controls are the transfection of an empty vector (-).

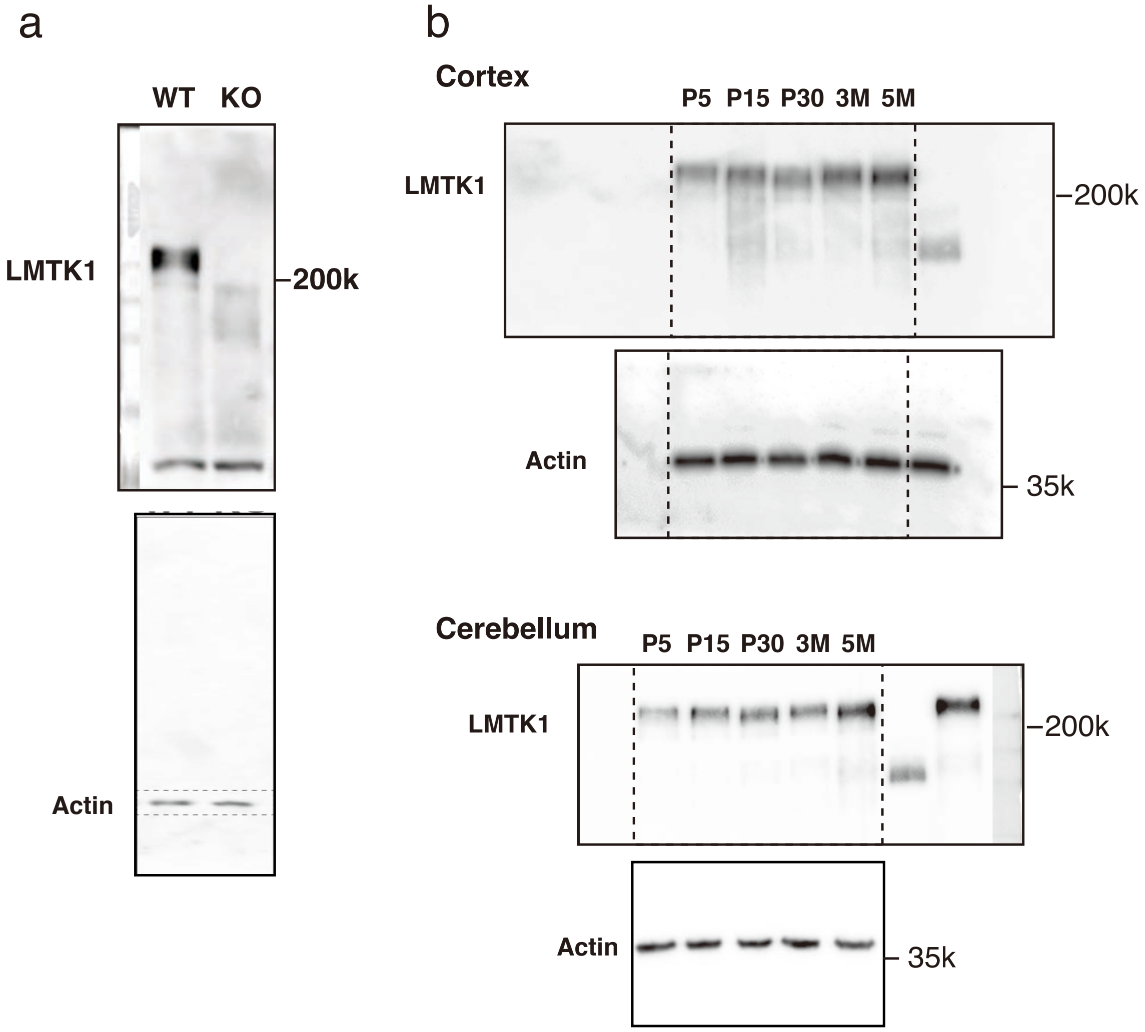

Supplementary Fig. 6. Original blots of Fig. 1.

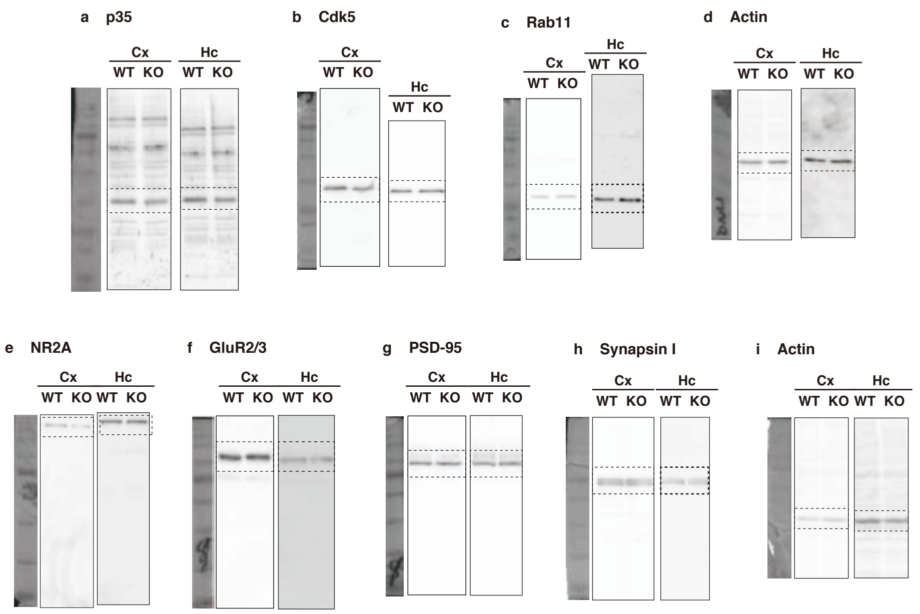

Supplementary Fig. 7. Original blots of Supplementary Figures 1 and 4. (a) ~ (d) Immunoblots of p35, Cdk5, Rab11 and actin in WT and LMTK1 KO mouse brains in Supplementary Fig. 1. (e) ~ (i) Immunoblots of NR2A, GluR2/3, PSD-95, synapsin I and actin in WT and LMTK1 KO mouse brains in Supplementary Fig. 4. The left side lane of each blot is molecular weight markers.
